# Research on Parameter Compensation Method and Control Strategy of Mobile Robot Dynamics Model Based on Digital Twin

**DOI:** 10.3390/s24248101

**Published:** 2024-12-19

**Authors:** Renjun Li, Xiaoyu Shang, Yang Wang, Chunbai Liu, Linsen Song, Yiwen Zhang, Lidong Gu, Xinming Zhang

**Affiliations:** 1 School of Mechanical and Electrical Engineering, Changchun University of Science and Technology, Changchun 130022, China; renjun.li@cust.edu.cn (R.L.); shangxiaoyu408@outlook.com (X.S.); gulidong@cust.edu.cn (L.G.); 2 Changchun Guanghua Microelectronic Equipment Engineering Center Co., Ltd., Changchun 130012, China; 3 FAW Foundry Co., Ltd., Changchun 130022, China; liuchunbai@faw.com.cn; 4 School of Mechanical Engineering and Automation, Foshan University, Foshan 528225, China; zxm@fosu.edu.cn

**Keywords:** digital twins, inspection robots, nonlinear dynamics, parameter compensation

## Abstract

Inspection robots, which improve hazard identification and enhance safety management, play a vital role in the examination of high-risk environments in many fields, such as power distribution, petrochemical, and new energy battery factories. Currently, the position precision of the robots is a major barrier to their broad application. Exact kinematic model and control system of the robots is required to improve their location accuracy during movement on the unstructured surfaces. By a virtual engine and digital twins, this study put forward a visualization monitoring and control system framework which can address the difficulties in the intelligent factories while managing a variety of data sources, such as virtual–real integration, real-time feedback, and other issues. To develop a more realistic dynamic model for the robots, we presented a neural-network-based compensation technique for the nonlinear dynamic model parameters of outdoor mobile robots. A physical prototype was applied in the experiments, and the results showed that the system is capable of controlling and monitoring outdoor mobile robots online with good visualization effects and high real-time performance. By boosting the positional accuracy of robots by 18% when navigating obstacles, the proposed precise kinematic model can increase the inspection efficiency of robots. The visualization monitoring and control system enables visual, digital, multi-method, and complete real-time inspections in high-risk factories, such as new energy battery factories, to ensure the safe and stable operations.

## 1. Introduction

A number of innovative tools and technologies have been applied in the manufacturing system with the introduction of Industry 4.0 and the growth of digital engineering [1], and the traditional manufacturing methods have been transferring to modernity and intelligence [2]. It is crucial for the new energy battery industry to enhance their safety risk examination to guarantee secure manufacturing because of the intricacy of their machinery and materials, which are hazardous, flammable, and explosive [3,4,5]. Manual inspection for potential risks has some issues, such as large labor loads, inefficiency, and low inspection quality [6]. Intelligent inspection robots that integrate informatization, digitization, networking, and intelligence can provide factory services, including timely interaction, autonomous decision-making, and system autonomy [7,8]. It has become popular to take advantage of inspection robots for real-time monitoring and intelligent management in high-risk environments. However, the control accuracy of these robots is inadequate, and the integrated monitoring data visualization presents challenges because of the relatively complex working environment.

According to their operational environments, mobile inspection robots have three categories, indoor, outdoor, and the newly suggested indoor–outdoor ones. The requirements for inspection robots are growing due to a scarcity of human resources [9]. The application scope of outdoor mobile robots is severely limited by problems like low positioning accuracy and poor stability arising from complicated and unpredictable working environments, which are characterized by irregular terrains [10]. The current challenges of the outdoor mobile robot development are to improve the positioning and control accuracy as well as address real-time monitoring and control issues [11].

Environmental awareness can improve robotic positioning accuracy significantly [12,13]. Robots operation can be smoother and more effective by modifying their control parameters and placement techniques in response to their perception of surroundings. Payam Nourizadeh et al. [14] proposed a method for estimating unexpected slippage in outdoor mobile robots. Sarcevic P, Qiu Z and Sango M et al. [15,16,17] proposed an outdoor-scene-understanding method for mobile robots to provide useful information for the control and navigation algorithms of wheeled mobile robots, as well as to increase the positioning accuracy indirectly. This method uses outdoor scene images to estimate the appropriate traversable area. By creating an algorithm that extracts vibration indication data from point clouds, Sathian Pookkuttath introduced a novel outdoor mobile robot state detection approach based on 3D LiDAR, achieving robot state identification at cheap computing costs [18]. Hanan A. Atiyah, Woo Seok Lee, and others [19,20,21,22,23,24] presented a number of environment-aware mobile-robot-positioning techniques, which reduced positioning difficulties in intricate situations and improved robot positioning accuracy.

Digital twinning is a high-fidelity simulation system that can imitate and affect real-world environments [25]. It allows for data to be shared offline or in real time with physical systems, which is a useful way to deal with real-time robotics monitoring and control problems [26]. Digital twinning technology enables the development of integrated systems across platforms and disciplines by integrating heterogeneous hardware and software components, which lays the groundwork for the transition to Industry 5.0 applications [27]. Yuan Zhao et al. [28] presented a closed-loop controlled digital twinning production line framework based on electromechanical integration techniques to solve the difficulties associated with data fusion, analysis, processing, and application in smart factories. Nils Kroell and others [29] merged IoT technology with machine learning and process control to create a data-driven digital twinning system that improved sorting plants’ accuracy and efficiency. Digital-twinning-based monitoring systems are characterized by high fidelity, synchronization, robust scalability, and extensive functionality, which improve facility-related data visualization and offer critical supervision for robotics greatly in workshops and automated factories. Hussen Sami Salama Hajjaj et al. [30] created a portable framework for human–robot interaction (HRI) for outdoor robots running the ROS system. This framework allows operators to monitor and manage mobile robots while doing autonomous tasks. In order to enable operators to monitor real-time robot operations using VR/AR and analyze operational data easily, Aschenbrenner et al. [31] separated sensor and robot motion data into distinct databases and visualized models and data with Unity. To offer highly visual real-time monitoring capabilities, Li et al. [32] suggested a semantically upgraded digital twinning monitoring system that focuses on multidimensional data gathering and processing. Zhou Yong et al. [33] presented a digital twinning-based platform for port crane monitoring to improve data display and acquisition transparency.

Considering the use of digital twins in the domains of industrial inspection and other fields, the two primary issues with the majority of the current studies on mobile robotic inspection systems are as follows:

(1) We require a dynamic model that can be applied to unknown situations in order to increase the positioning accuracy of outdoor mobile robots [34,35]. However, since the mobile robot is a complicated multi-DOF system, it is challenging to create an accurate dynamic model which depends on a variety of elements, including the slope and smoothness of the road surface [36].
(2) No platforms for robotic surveillance systems can be customized to fit different application situations, features, controls, visualization, and other needs [37].

To achieve the same dynamic properties as the physical model, this study suggests a digital-twin-based control framework for a mobile inspection robot. This framework takes advantage of digital twin technology to create a model of the mobile robot and train it in a virtual environment. To increase the control precision of the robot, the control system uses the dynamic model created by the digital twin system. In order to solve issue (1), this paper proposes a parameter compensation method and control strategy for a mobile robot dynamics model based on a digital twin. Through the digital twin technology, a nonlinear inverse dynamic model of the 4WD mobile robot is established, and the relevant parameters in the dynamic model of the mobile robot are obtained, which improves the accuracy of the dynamic model. To answer issue (2), this article creates an integrated platform for monitoring and control by utilizing a virtual engine to create a visual monitoring and control system based on a digital twin. This allows for information interaction and integration between the monitoring and control systems. Furthermore, this paper integrates health management functions of the inspected plant into the surveillance and control systems. This allows for the operators to oversee the work between processes within the factory under a single interface, thereby easing the processing of multiple source data, virtual, and real-time merging feedback issues in the smart factory.

This paper was structured as follows: in Section 2, a digital-twin-based control strategy and visual monitoring and control system for mobile robots is proposed; in Section 3, the process for creating nonlinear dynamic models and parameter compensation techniques for mobile robots is explained; in Section 4, the feasibility and efficacy of the suggested approach are confirmed through experiments; and in Section 5, conclusions, implications, limitations, and future research directions are suggested.

## 2. Visual Monitoring and Control System for Inspection Robots

### 2.1. System Architecture for Inspection Robots Based on Digital Twin

**Figure 1:**
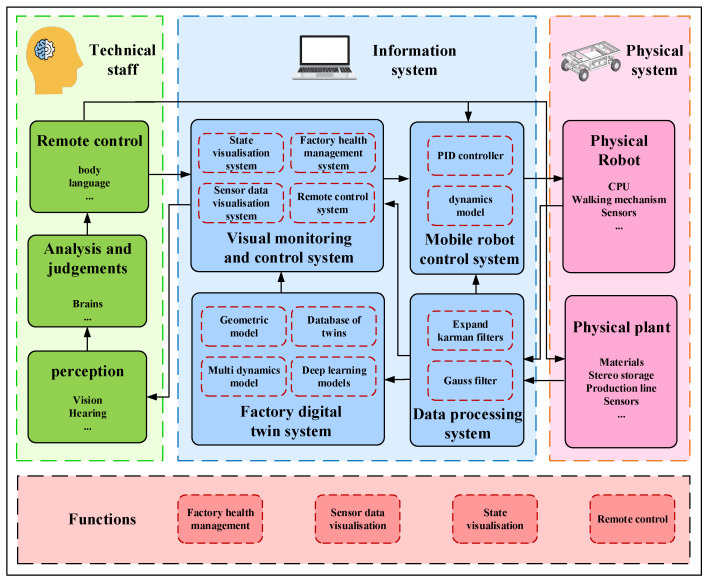
Composition framework for mobile robotic surveillance systems based on digital twins.

The composition framework of the mobile robot inspection system based on digital twin proposed in this article is shown in Figure 1. In a physical system, sensors gather information about the condition of the mobile robot and the surrounding manufacturing environment, then send it over Industrial Ethernet to the visual monitoring and control system. The information system improves the factory operational safety by providing technicians with quick access to pertinent information through the creation of a data-processing system, a digital twin system, and a visual monitoring and control system. Then, the technicians receive the pertinent information promptly through the visual monitoring and control system. By interacting with the information system in real time, technicians find out how factories and inspection robots are operating. If needed, they can even manage the inspection robot using their operational expertise remotely.

A mirror image of the physical entity is created in the virtual space by the digital twin system, which also analyzes the sensor data collected from the factory and the mobile robot, such as temperature, pressure, depth camera, and IMU data, performs health checks on the factory operational status, and displays the detection data visually. The digital twin ensures the reliable operation of systems and equipment by allowing technicians to see the condition of inspection robots and equipment in real time. The digital twin system sends the proper motion parameters to the physical entity of the mobile robot to achieve the compensation of the dynamic model parameters of the mobile robot.

Equipment performance, health, and operating conditions are tracked and assessed through the application of factory health management, aiding manufacturers in preventing equipment failures and shutdowns by providing early failure alerts. In order to achieve synchronous scheduling and operational planning of mobile robotic equipment and production equipment, as well as to improve production efficiency and product quality, sensor data can be represented graphically. Through the use of physical inspection robot data, state visualization creates digitalized models by mapping and updating the movement paths of the robots. This allows for the visual representation of both the factory operations and the inspection robot states of movement.

### 2.2. Digital Twin System for High-Risk Factories

#### 2.2.1. Digital Twin System Architecture

Greves et al. [38] suggested the notion of digital twins and three-dimensional structures in 2003 initially. They described the three layers of physical entities, virtual entities, and data links that a digital twin system must contend with. The domains of aircraft design, workshop management, stereo warehouse maintenance, etc., have all benefited from the deployment of digital twin technology due to the growth of linked technologies and the growing need for applications. Digital twins still have issues though, like lacking universality; inability to create dynamic, multidimensional time–space-scale models; and inability to update data at the right time. A five-dimensional space model for the digital twin system was later introduced by Tao Fei et al. [39,40]. Building on the foundation of the original model, the five-dimensional spatial model of the digital twin system added two dimensions of service and twin data, thereby enhancing the model’s application.

The five-dimensional space concept of the digital twin expressed mathematically in Formula (1):

*MDT* = (*PE*, *VE*, *SS*, *DD*, *CN*) (1)

*PE* stands for physical entity, *VE* for virtual entities, *SS* for services, *DD* for twin data, and *CN* for connections between components in the formula.

Sebastian Richard Newrzella et al. [41] presented a five-dimensional cross-industry digital twin application model to facilitate the understanding of cross-industry applications as well as digital twin applications. Chunlong Wu et al. [42] suggested a five-dimensional digital twin framework for the modeling approach to depict the intricate link between the digital duplicate item and its properties.

The challenges that digital twins in the aforementioned models must overcome include data acquisition and integration, multidisciplinary consolidation, model accuracy and accurateness, computational resource requirements, security and privacy concerns, performance verification and real-time updates, model application, and cost-effectiveness [43,44,45]. So, to overcome these obstacles, we need comprehensive planning and management as well as the coordinated use of knowledge and technology across multiple sectors.

#### 2.2.2. Digital Twin System for Mobile Robots

The absence of a universal design model standard is now one of the issues with the implementation of a mobile robot digital twin system [46]. This paper presents a mobile robotic digital twin system within the five-dimensional framework of the digital robot twin. The system is primarily composed of the mobile robot character entity, mobile robot virtual entities, mobile robotics twin databases, mobile robots service management system, and the connections between different parts.

A digital twin system is built on physical entities, which primarily consist of industrial computers, routers for data exchange, sensors, mobile robots, and inspection tools. The essential component of the digital twin system is the virtual entity, which simulates the physical entities to represent the mobile robot actual movements and interactions with the inspection vehicle. Data from physical entities, virtual entities, and service applications are stored in the twin database for mobile robots. These data include sensor information, control data, operational status data, motion data of the virtual mobile robot model, and the task data from the mobile robot past. Two categories of services—one geared toward physical things and the other toward virtual entities—are distinguished inside the mobile robot service management system. The primary services targeted at physical entities are task dispatching, energy consumption optimization, status prediction, and monitoring. The main services focused on virtual entities are testing, calibration, and model building. To facilitate data interactions for complicated activities, the five-dimensional digital twin system has bidirectional links between physical entities, virtual entities, the twin database, and the service management system.

The mobile robot sensor data are uploaded wirelessly to the digital twin system twin database in this setup. The virtual entity calculates each wheel motion error, drives the twin model motion, collects sensor data from the database, and uploads the results back to the digital twin database. The wheel motion mistakes and the robot current motion status are sent to the mobile robot control system. It modifies state and sends the motion parameters to the mobile robot physical entity based on the target motion, achieving the compensation of the dynamic model parameters and enhancing the precision of the mobile robot control.

### 2.3. Design of Visual Monitoring and Control System

**Figure 2:**
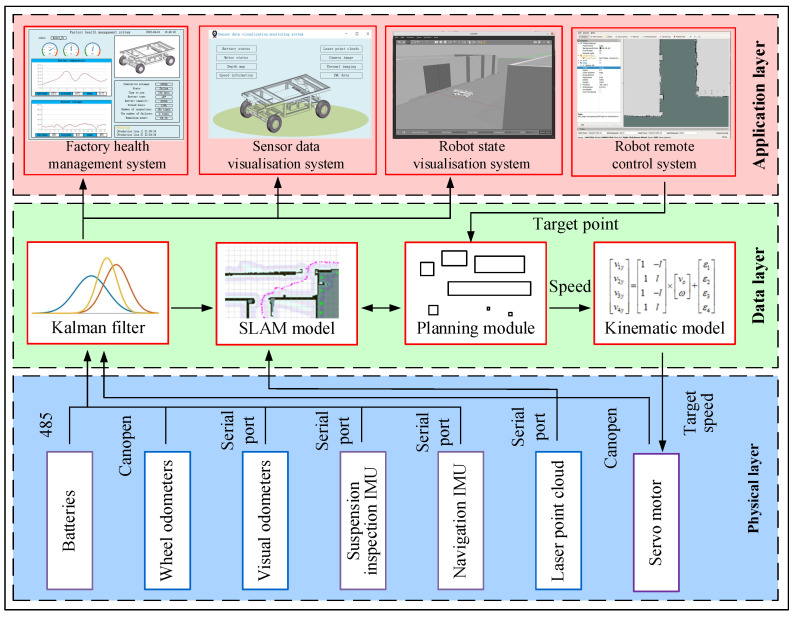
Visual monitoring and control system.

The advanced management system known as the visual monitoring and control system makes use of virtual reality technology to provide three-dimensional visual monitoring as well as abnormal monitoring of the robot status, working environment, and sensor data while moving. By digital twin mapping linking to real mobile robots, processing equipment, and other devices, it is possible to monitor and operate mobile robot equipment in several dimensions. As seen in Figure 2, the primary system components include a factory health management system, a robot status visualization display system, a sensor data visualization display system, and a remote robot control system.

One of the most important technologies to guarantee a factory regular operation is the health management system, an application that tracks and assesses the operational status and health of a factory [47]. To determine whether the equipment status data of mobile robots and inspection factories are within a tolerable range, this article gathers, detects, and analyzes the data, which improves the factory maintenance efficiency by identifying anomalous situations quickly within the facility as well as anomalies within the robots.

The sensor data visualization display system makes it possible to monitor sensor data, including voltage, current, and stereo camera data. Visual monitoring of the sensor-collected data is achieved by publishing the data of physical layer in a standard topic structure to the RViz platform, which then presents it graphically. This enhances production efficiency and product quality by facilitating the coordinated scheduling and operational planning of mobile robot and manufacturing equipment.

The robot status visualization display system is an application that enables visual monitoring of the state and working environment of mobile robots. First, it gathers real-time status data from the physical environment, including the mobile robot’s IMU, servo motor torque, speed, and battery information. The information obtained, including the workstation status of the robot, the rotation speed of each wheel, and the independent suspension status, is processed and analyzed by the data-processing module in the data layer. It is then published to the RViz visualization platform in the form of ROS standard topics. This real-time driving of the robot model makes it possible for real-time visual monitoring of the robot motion status. Operators can view the mobile robot equipment position, operational status, and motion trajectory in real time in the status visualization scenario. The robot remote-control system makes it possible for real-time monitoring and scheduling, which allows operators to operate and command the mobile robot remotely at any time.

Robots can be operated and controlled from a distance thanks to robot remote-control systems [48], which lay the groundwork for autonomous locomotion. In recent years, study interest in the robot remote-control system based on multi-sensor fusion technology is growing [49]. This article uses multi-sensor fusion technology to combine and evaluate several sensor data types, including depth camera and lidar, in the mobile robot system. This helps the inspection robot perceive its surroundings more clearly and comprehend outside situations more deeply. The ground station PC terminal manages the mobile robot remotely over the local area network, which increases the robot’s adaptability to challenging conditions and boosts the precision and dependability of the control system.

## 3. Nonlinear Dynamics Parameter Compensation Strategy

We cannot deduce kinematics from theoretical formulas for a mobile robot since it is a complicated multi-degree-of-freedom system whose dynamic model is nonlinear and evolves with the changes in the robot operational condition. A two-dimensional plane model is typically used to represent the dynamic model when a mobile robot operates in an organized road environment. Nevertheless, this technique is not suitable for unstructured settings, leading to subpar motion accuracy in those situations.

### 3.1. Nonlinear Inverse Dynamics Model for Mobile Robots

**Figure 3:**
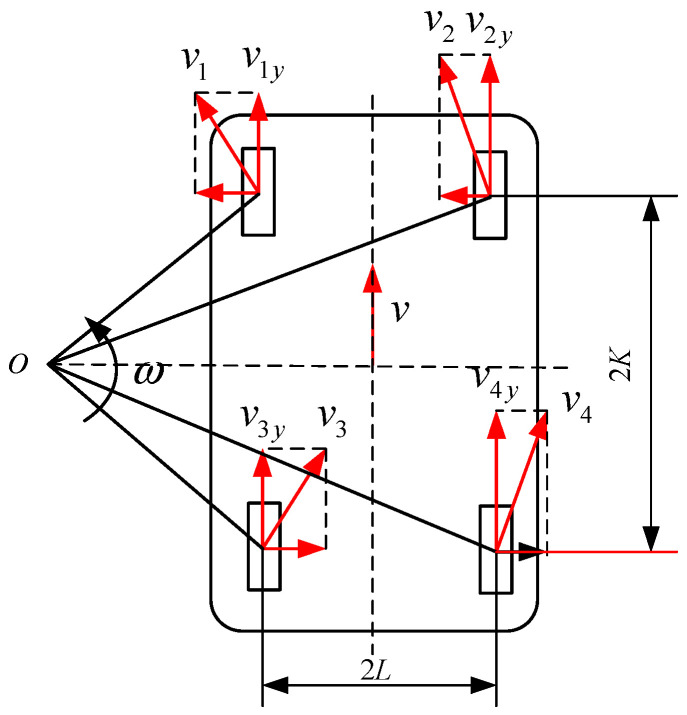
Simplified dynamics model for mobile robots.

The dynamic model of a four-wheel-drive differential steering mobile robot can be simplified into a dynamic model in two-dimensional space, as shown in Figure 3, by ignoring the nonlinearity of the mobile robot and the impact of variables like changes in road conditions on the robot movement. This is assuming that the centroid of the car is the mass center.

This Figure shows that the wheelbase between the front and rear wheels is 2*K*, the distance between the left and right wheels is 2*L*, and the linear and angular velocities of the vehicle centroid are *v*, respectively. The wheel centers real traveling directions are represented by the numbers *V*_1_, *V*_2_, *V*_3_, and *V*_4_, while their projections along the *y*-axis are shown by the numbers *V*_1*y*_, *V*_2*y*_, *V*_3*y*_, and *V*_4*y*_.

Each wheel *y*-axis component velocity is as follows:

(2) $$\left[\begin{array}{c} v_{1y}\\ v_{2y}\\ v_{3y}\\ v_{4y} \end{array}\right]=\left[\begin{array}{c} \begin{array}{cc} 1 & -l \end{array}\\ \begin{array}{cc} 1 & l \end{array}\\ \begin{array}{cc} 1 & -l \end{array}\\ \begin{array}{cc} 1 & l \end{array} \end{array}\right]\times \left[\begin{array}{c} v_{o}\\ \omega \end{array}\right]$$

The *y*-axis component velocity of each wheel on the mobile robot can be represented as follows when taking into account how the road surface affects the dynamic model of the mobile robot:

(3) $$\left[\begin{array}{l} v_{1y}\\ v_{2y}\\ v_{3y}\\ v_{4y} \end{array}\right]=\left[\begin{array}{cc} 1 & -l\\ 1 & l\\ 1 & -l\\ 1 & l \end{array}\right]\times \left[\begin{array}{l} v_{o}\\ \omega \end{array}\right]+\left[\begin{array}{l} \varepsilon_{1}\\ \varepsilon_{2}\\ \varepsilon_{3}\\ \varepsilon_{4} \end{array}\right]$$

(4) $$\varepsilon ={\left[\begin{array}{c} \varepsilon_{1}\;\;\;\;\varepsilon_{2}\;\;\;\;\varepsilon_{3}\;\;\;\;\varepsilon_{4} \end{array}\right]}^{T}$$

where ε represents the influence of unstructured road environments on the dynamics model of the mobile robot.

However, physical rules and theories relating to dynamics cannot be used to solve ε [50]. To gather the motion data of the inspection robot in the digital twin model and use machine learning to estimate ε, this research considers a data-driven strategy.

### 3.2. Strategy for Compensating Parameters in a Nonlinear Dynamics Model

**Figure 4:**
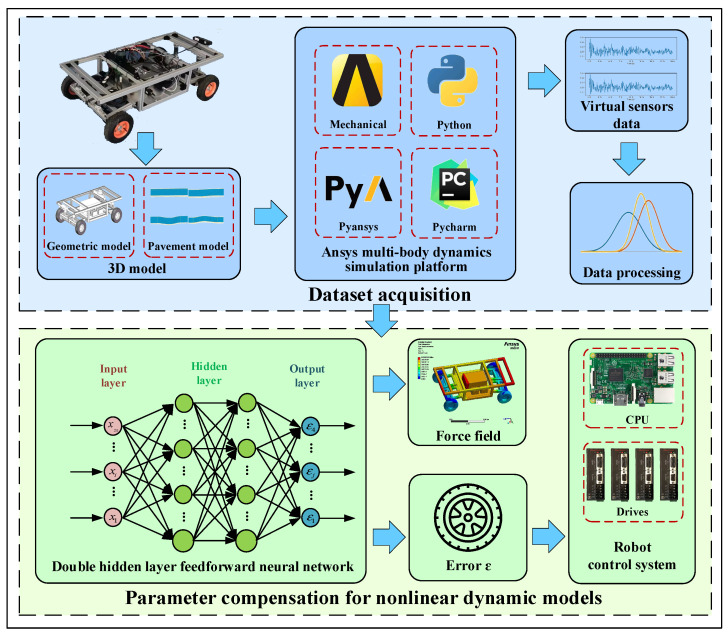
The method for compensating parameters in a nonlinear dynamic model.

Figure 4 illustrates the two steps in the process to adjust parameters in a nonlinear dynamic model: acquiring the information and building the dynamic model.

#### 3.2.1. Acquisition of Motion Datasets

A mobile robot construction can be divided into several tiny components using finite element technology, which makes the robot flexible and discretizes the mathematical model. This makes it possible to solve partial differential equations and determine, while accounting for deformation, the motion information of every mobile robot component during real-world motion. In this study, we secondarily developed the Ansys program using Python 3.10. A dynamic model of the mobile robot is built based on finite element technology, and datasets are obtained for the motion of the mobile robot under various operating situations.

This article discusses the application of a mobile inspection robot in both indoor and outdoor conditions. In practical applications, we must navigate uneven road surfaces including bumps, incline, and red brick roads, necessitating the mobile robot to have a specific capacity to navigate obstacles. In this article, we modeled numerous typical road surfaces, such as flat roads, concave roads, convex roads, and complex roads, using SolidWorks 2021 to mimic the motion states of various mechanisms during the operation of the mobile robot over non-structured road surfaces.

After meshing the road surface and mobile robot on the Hypermesh platform, ANSYS 2021R1 was used to run simulations under various operating conditions. These conditions fell into four main categories: turning acceleration, straight constant speed, straight acceleration, and turning constant speed. There were 92 distinct working circumstances overall, with subcategories based on variations in movement speed, acceleration, and road surface types within each main group. Table 1 displays each condition constraint conditions. After calculations, the motion datasets of the mobile robot based on the digital twin were obtained by Python to extract the virtual sensor information from the dynamic model and write it into the text file.

#### 3.2.2. Calculation and Compensation of Dynamic Model Errors

Neural networks, which can fit complex nonlinear functions, are able to explain a great deal of previously inexplicable nonlinear functions, mainly because ε is highly nonlinear and cannot be represented using theoretical formulas. This paper establishes a nonlinear dynamic model for the mobile robot by neural networks to find the relationship between ε and the robot motion parameters.

The ANSYS dynamics model value of ε is computed following the acquisition of the mobile robot motion datasets. The following is the formula used to calculate ε:

(5) $$\varepsilon =\left[\begin{array}{c} v_{1y}\\ v_{2y}\\ v_{3y}\\ v_{4y} \end{array}\right]-\left[\begin{array}{c} \begin{array}{cc} 1 & -l \end{array}\\ \begin{array}{cc} 1 & l \end{array}\\ \begin{array}{cc} 1 & -l \end{array}\\ \begin{array}{cc} 1 & l \end{array} \end{array}\right]\times \left[\begin{array}{c} v_{o}\\ \omega \end{array}\right]$$

Following the computation of ε, a dataset with the following parameters is obtained:

Among them, *h_ix_*(*i* = 1, 2, 3, 4), *h_iy_*(*i* = 1, 2, 3, 4), and *h_iz_*(*i* = 1, 2, 3, 4) represent the IMU sensor data at the *i*th suspension point.

The following is the relationship between $\varepsilon_{t}$ and *v_t_*, *v_t_*_−1_, *θ_t_*, *θ_t_*_−1_, *h^ix^_t_*(*i* = 1, 2, 3, 4), *h^iy^_t_*(*i* = 1, 2, 3, 4), *h^iz^_t_*(*i* = 1, 2, 3, 4), *h^ix^_t_*_−1_(*i* = 1, 2, 3, 4), *h^iy^_t_*_−1_(*i* = 1, 2, 3, 4), and *h^iz^_t_*_−1_(*i* = 1, 2, 3, 4) using a backpropagation neural network for training:

(6) $$\begin{array}{l} \varepsilon_{t}=f(v_{t},\theta_{t},{h^{1x}}_{t},{h^{1y}}_{t},{h^{1z}}_{t},{h^{2x}}_{t},{h^{2y}}_{t},{h^{2z}}_{t},{h^{3x}}_{t},{h^{3y}}_{t},{h^{3z}}_{t},\\ \;{h^{4x}}_{t},{h^{4y}}_{t},{h^{4z}}_{t},v_{t-1},\theta_{t-1},{h^{1x}}_{t-1},{h^{1y}}_{t-1},{h^{1z}}_{t-1},{h^{2x}}_{t-1},\\ \;{h^{2y}}_{t-1},{h^{2z}}_{t-1}{h^{3x}}_{t-1},{h^{3y}}_{t-1},{h^{3z}}_{t-1},{h^{4x}}_{t-1},{h^{4y}}_{t-1},{h^{4z}}_{t-1}) \end{array}$$

where $\varepsilon_{t}$ represents the effect of the road surface on the dynamics model of the mobile robot at time *t*. *v_t_* and *θt* refer to the linear velocity and angular velocity of the center of the mobile robot at time *t*. ${h^{1y}}_{t},{h^{1z}}_{t},{h^{2x}}_{t},{h^{2y}}_{t},{h^{2z}}_{t},{h^{3x}}_{t},{h^{3y}}_{t},{h^{3z}}_{t},{h^{4x}}_{t},{h^{4y}}_{t},{h^{4z}}_{t}$ represent the IMU sensor data at the four suspension points at time *t*, while *v_t−1_* and *θ_t_*_−1_ refer to the linear velocity and angular velocity of the center of the mobile robot at time *t* − 1. ${h^{1x}}_{t-1},{h^{1y}}_{t-1},{h^{1z}}_{t-1},{h^{2x}}_{t-1},{h^{2y}}_{t-1},{h^{2z}}_{t-1}{h^{3x}}_{t-1},{h^{3y}}_{t-1},{h^{3z}}_{t-1},{h^{4x}}_{t-1},{h^{4y}}_{t-1},{h^{4z}}_{t-1}$ represent the IMU sensor data at the four suspension points at time *t* − 1.

A double-hidden-layer feedforward neural network with 28 input neurons, 4 output neurons, and 40 hidden-layer neurons makes up the neural network that was chosen. As the activation function, the sigmoid function is employed, and the error objective function is as follows:

(7) $$E_{k}=\lambda \frac{1}{m}\sum_{k=1}^{m}E_{k}+(1-\lambda )\sum_{i}\omega_{i}^{2}$$

where $E_{k}$ stands for the connection weights and thresholds and reflects the error of the *k*th training sample. In order to estimate, the cross-validation approach is applied, with the goal to balance network complexity and empirical error.

The neural network model is trained using the gradient descent approach on the TensorFlow 2.15 platform. This yields the error ε in the nonlinear dynamic model of the mobile robot based on digital twin technology.

## 4. Experiment

An experimental prototype of an indoor–outdoor mobile inspection robot was built to verify the pertinent features of the digital twin-based visual monitoring and control system for mobile robots as well as the efficacy of the suggested nonlinear dynamics model parameter compensation method for mobile robots. Experiments were carried out on the virtual–real consistency, visual monitoring and control, and positioning accuracy of the mobile robot within the setting of a new energy battery production factory.

### 4.1. Physical Platform of Mobile Robot

**Figure 5:**
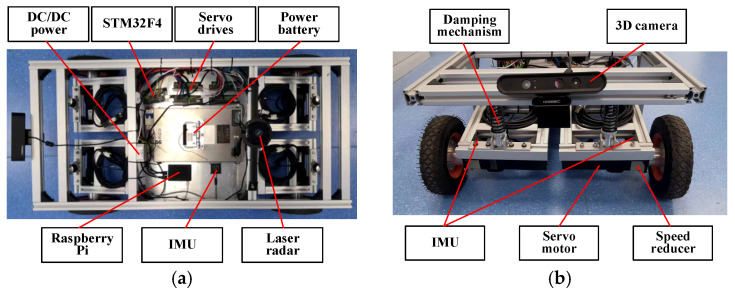
Multi-sensor physical platform of a mobile robot: (**a**) top view; (**b**) front view.

One of the most important technological advancements for increasing robot positioning accuracy is multi-sensor fusion positioning. The inadequacies of individual sensors are accounted for by combining data from visual odometry, laser positioning, and GPS positioning, leading to a satisfactory location estimate for the robot [51,52]. A multi-sensor physical platform for the mobile robot was constructed, as illustrated in Figure 5, in order to verify the viability of the digital-twin-based mobile robot control system suggested in this study.

The mobile robot assembled in this study has a four-wheel drive (4WD) chassis. Each wheel is powered with four servo motors and a swing-type independent suspension system. So, the robot can adjust its movement against different types of road surfaces. The lower computer of the mobile robot is an STM32F4 development board (manufactrued by EMT Co., Ltd from Guangzhou, China), and the upper computer is a Raspberry Pi 4B (packaged together with STM32F4 development board). Under the ROS 2 (Robot Operating System 2) system architecture, the mobile robot control system was developed. The essential specifications of the fundamental parts of mobile robots are displayed in Table 2.

**Figure 6:**
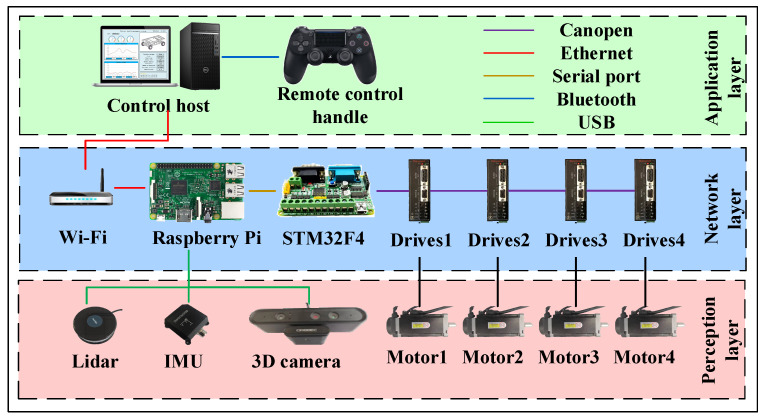
Control system architecture diagram of a mobile robot based on digital twin.

The three levels that make up the architecture of the digital-twin-based mobile robot control system are the perception layer, the network layer, and the application layer, as illustrated in Figure 6. The perception layer uses devices such a lidar, IMU (Inertial Measurement Unit), depth camera, and servo motors to gather information about the mobile robot exterior surroundings. Data transmission between different modules is made possible by the network layer, which sends the external environmental data that the perception layer has recorded to the application layer. The primary parts of it are servo drivers, an STM32F4, a Raspberry Pi, and a WIFI module.

The application layer gives users an interactive interface to monitor and operate the robot in addition to acting as the platform for running programs. A control computer, a joystick for remote control, and additional parts make up its main parts. Different information-gathering programs are built and communicated via TCP/IP protocols to the ground control station of the mobile robot, depending on the various protocols used for sensor communication. Next, the status, monitoring, and task assignment of the mobile robot are visualized using digital twin technology.

### 4.2. Virtual–Real Consistency Experiment

**Figure 7:**
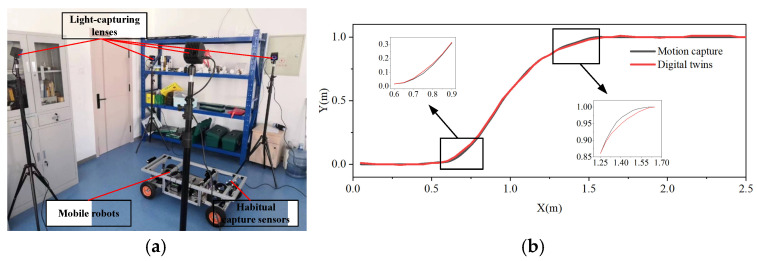
Experiment on the consistency between the virtual and real mobile robot: (**a**) test scenario; (**b**) experimental result curve.

A virtual–real consistency experiment was carried out to quantify the positional error between the mobile robot in the visualization monitoring system and the mobile robot in the real world. Figure 7a depicts the experimental scene. The mobile robot is instructed to begin at the coordinate (0, 0) and move forward indoors at a steady speed of 1 m/s. Once it has reached the coordinate point (0.6, 0), it is instructed to move to the coordinate (1.6, 1) and then continue moving forward. The mobile robot position point information is measured using a motion capture system that combines inertia and optics. The digital twin system then extracts the mobile robot previous position point coordinates. Figure 7b displays the findings of the mobile robot virtual–real consistency experiment.

This graph plots the relative positions over time between the positions obtained by the visualization-monitoring system (virtual positions) and the positions measured by the motion capture system (real-world positions). The degree of consistency between the virtual and physical representations of the mobile robot is shown by how similar the two curves are.

The largest positional inaccuracy between the virtual and real-world models of the mobile robot is 2 mm, as Figure 7b makes evident, demonstrating the high degree of consistency between the two. The influence of the robot-positioning algorithm and sensor accuracy causes an increase in the positional error between the virtual and real-world models when the mobile robot turns abruptly. The movement of the model in the digital twin lags behind the real-world model by about 160 ms because of the processing resources needed to solve the model pose in the digital twin and the time required for signal transmission. On the other hand, the experimental results show that the suggested digital-twin-based mobile robot control system can offer real-time viewing and monitoring features, allowing users to comprehend the mobile robot state and behavior. This eases the task of controlling mobile inspection robots greatly.

### 4.3. Visualization Monitoring and Control Experiment

**Figure 8:**
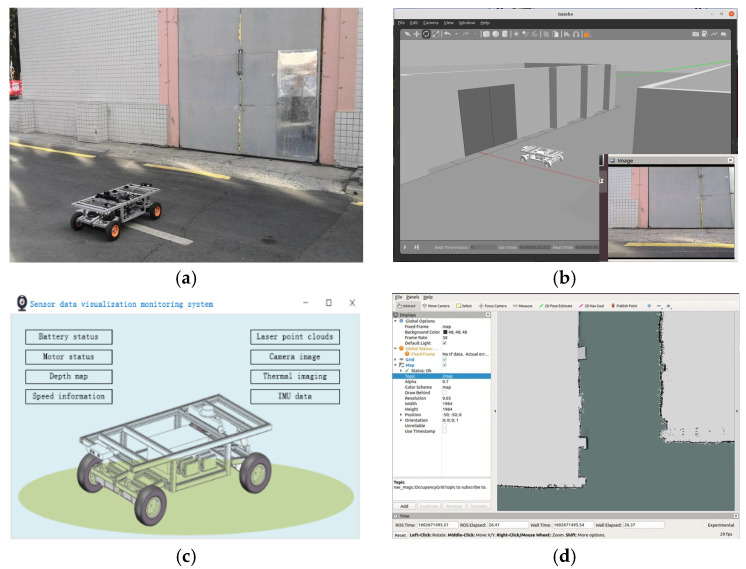
Experiment scenario and results of visual monitoring and control: (**a**) test scenario for visual monitoring and control experiment; (**b**) interface of the status visualization system; (**c**) interface of the sensor data visualization system; (**d**) interface of the remote robot control system.

This Section examines the functionality of each platform module based on the mobile robot control strategy and its visualization monitoring system. Figure 8 displays the test scenario and outcomes.

The trials have shown that users can inspect the state of the factory motors, materials, and equipment remotely thanks to the robot status visualization system. Users can monitor the mobile robot battery status, motor status, speed, laser point cloud, camera images, IMU data, and other information by utilizing the sensor data visualization system. With the remote robot control system, users can direct the mobile robot to go to designated areas and monitor the condition of related equipment. The system modules can cooperate effectively to produce coordinated scheduling and operation planning for mobile inspection robots and manufacturing equipment, as demonstrated by the visualization-monitoring experiments, which have validated the functionality of each module.

The trial findings additionally demonstrate the ability of the digital-twin-based visual monitoring and control system described in this study to process and integrate data in new energy battery factories, enabling the factory to be monitored in real time by visualization technologies. The practical requirements of high-risk industries like new energy batteries, petrochemicals, and power transmission and distribution are better served by this monitoring technique. This method helps to improve the inspection and production efficiency of new energy factories, decrease production downtime, and identify and fix equipment issues quickly. It guarantees the stable and safe operation of factories with a high risk to businesses while also cutting personnel costs.

### 4.4. Positioning Accuracy Experiment

**Figure 9:**
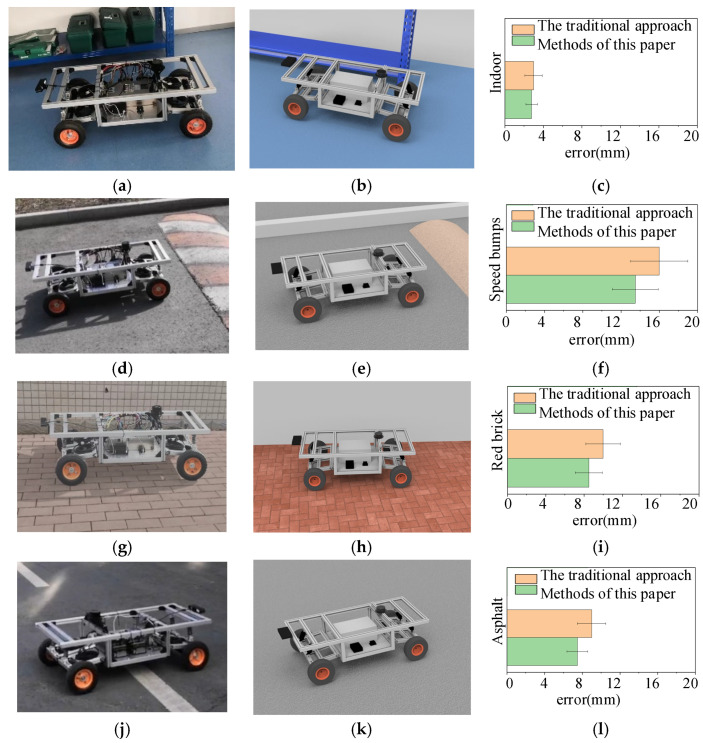
Experimental scenarios and results of positioning accuracy: (**a**) indoor pavement experimental test scenario; (**b**) digital twin of indoor pavement; (**c**) experimental errors on indoor pavement; (**d**) deceleration strip pavement experimental test scenario; (**e**) digital twin of deceleration strip pavement; (**f**) experimental errors on deceleration strip pavement; (**g**) red brick pavement experimental test scenario; (**h**) digital twin of red brick pavement; (**i**) experimental errors on red brick pavement; (**j**) asphalt pavement experimental test scenario; (**k**) digital twin of asphalt pavement; (**l**) experimental errors on asphalt pavement.

Using a variety of road conditions, such as indoor smooth pavement, outdoor deceleration strip pavement, outdoor red brick pavement, and outdoor asphalt pavement, positioning accuracy experiments were carried out to verify the efficacy of the dynamics model parameter compensation method based on digital twins presented in this paper. Figure 9 displays the experimental situation and outcomes. Throughout the experiment, a remote-control system was utilized to provide commands to the robot, and an optical and inertial motion capture system was employed to record the robot journey and obtain the coordinates of its path points. The average error and standard deviation of the mobile robot under various road conditions were then obtained by comparing these coordinates with the coordinates provided by the control instructions.

Figure 9 illustrates how, in comparison to the original method, the mobile robot controlled by the method suggested in this study has lower average and variance positioning errors under four distinct road conditions. This research proposes a strategy that lowers the positioning error by roughly 6% in an indoor smooth pavement environment and by approximately 18% in an outdoor unstructured-road environment. The results of the studies demonstrate that the dynamics model parameter compensation approach based on digital twins for mobile robots lower the robot-positioning inaccuracy successfully. The positioning accuracy of mobile robots is enhanced by this technology significantly, and the impact is particularly noticeable under unstructured road conditions. It is crucial for increasing the precision and dependability of the control system and the ability of mobile robots to adapt to complicated situations.

## 5. Conclusions

The mobile robots often experience lower positioning accuracy and stability when traversing uneven terrain due to the unstructured road surfaces in outdoor environments. This paper investigates the establishing methods of a visual monitoring and control system for mobile robots and the construction methods of nonlinear dynamics models. The working environments for indoor and outdoor inspection robots are complex, requiring an integrated platform that realizes the visual display of robots and their work environments, as well as robot control.

(1) A control approach for both indoor and outdoor inspection robots is proposed, based on a five-dimensional spatial model of a digital twin system. Sensor data from the mobile robot are uploaded to the twin database via a wireless network. By evaluating the robot operational state and sending the proper motion parameters to the physical mobile robot entity, the approach presented in this study is more practical than the current ones for controlling the past and present operating states of factories and mobile robots. It also increases the accuracy of mobile robot control and broadens the scope of applications of mobile robots.
(2) A visual monitoring and control system for indoor and outdoor inspection robots has been established to achieve three-dimensional visual monitoring and abnormal monitoring of the robot status, operating environment, and sensor information during the inspection process. Virtual reality technology is used in this system. This makes it possible to monitor mobile robots and industrial machinery in several dimensions visually. The usability and inspection efficiency of the mobile robots have been enhanced by integrating the visual monitoring system, factory health management system, and remote control system onto a single platform. This ensures the stable and safe operation of high-risk factories. In contrast to Yong Zhou’s [33] approach, this paper combines the visual monitoring system, factory health management system, and mobile robot remote control system into a single platform. This approach has a higher level of system integration, enhances system usability, and ensures the stable and safe operation of high-risk factories.
(3) When operating on complicated road surfaces, mobile robots jitter and have low precision due to the inaccuracy of linear models, which are used in the majority of current robot dynamics models. Thus, for the nonlinear inverse dynamic model of indoor and outdoor inspection robots, this work suggests a parameter compensation technique. We construct a multi-degree-of-freedom dynamics model for the mobile robot using digital twin and finite element technologies. A data-driven method is used to estimate the unknown parameters in the dynamics model by collecting motion data from the mobile robot under different pavement conditions, such as level pavement, complicated pavement, concave pavement, and convex pavement. This increases the stability and positioning accuracy of outdoor mobile robots by improving the accuracy of the mobile robot dynamics model.
(4) Through coordinated scheduling and operation planning for the mobile robot and production equipment, experiments on the mobile inspection robot have demonstrated the interoperability of the various modules of the visual monitoring and control system. This has allowed for the visual inspection in high-risk factories. Reducing positioning errors is a significant effect of the parameter compensation approach for the mobile inspection robot dynamics model, especially under unstructured road circumstances.

As a reference for its use in various digital twin scenarios, the approach suggested in this study will be applied in future work when large-scale data interaction occurs, optimizing data transmission stability, latency, CPU operation, and storage consumption. This study offers a solution to improve the positioning accuracy and stationarity of indoor and outdoor mobile robots. However, the method model in this paper is complex, and the control system requires a lot of computational resources to obtain an accurate dynamic model with sufficient accuracy. Therefore, future research can concentrate on simplifying the nonlinear dynamics model so that it can be used in a setting with constrained computational resources.

## Figures and Tables

**Table 1:** Operating conditions of mobile robots in the twin model.

Category	Constraints
go straight and start straight	Pavement: flat pavement, concave pavement, raised pavement, complex pavement; Speed: 0–1 m/s; Acceleration: 0.2 m/s^2^, 0.4 m/s^2^, 0.6 m/s^2^, 0.8 m/s^2^ and 1 m/s^2^.
straight at a constant speed	Pavement: flat pavement, concave pavement, raised pavement, complex pavement; speed: 0.2 m/s, 0.4 m/s, 0.6 m/s, 0.8 m/s, 1.0 m/s.
turn and start	Pavement: flat pavement, concave pavement, raised pavement, complex pavement; Speed: 0–1 m/s; Acceleration: 0.2 m/s^2^, 0.4 m/s^2^, 0.6 m/s^2^; Turning radius: 1 m, 2 m, 3 m.
constant speed in turning	Pavement: flat pavement, concave pavement, raised pavement, complex pavement; Speed: 0.2 m/s, 0.4 m/s, 0.6 m/s; Turning radius: 1 m, 2 m, 3 m.

**Table 2:** The essential specifications of the fundamental parts of mobile robots.

Part	Parameter	Value
3D camera	resolution Frame rate	1920 × 1080 30 fps
Lidar	Ranging principle Measure the radius Frequency of scans	TOF 30 m 10 Hz
Raspberry Pi 4B	CPU Running memory operating system	1.5 GHz 4 cores 8 GB Ubuntu 22.04
IMU	Number of axes Output frequency	9 100 Hz

## Data Availability

Data are contained within the article.
